# Supplementary material for: A methodology for the design of experiments in computational intelligence with multiple regression models
Source: PeerJ. 2016 Dec 1;4:e2721. doi: 10.7717/peerj.2721 (PMC5136129; doi:10.7717/peerj.2721)
Supplement: Data S1 — Datailed results from UC Irvine Machine Learning Repository (Housing, Machine CPU, Wine Quality, Automobile and Parkinson) and the 3 Use Cases (Protein Corona, Gajewicz Metal Oxides and Aquatic Toxicity) [file peerj-04-2721-s001.zip › 6_UseCases/2_GajewiczMetalOxides/RF.details.csv.repeatedcv.split3.pdf]

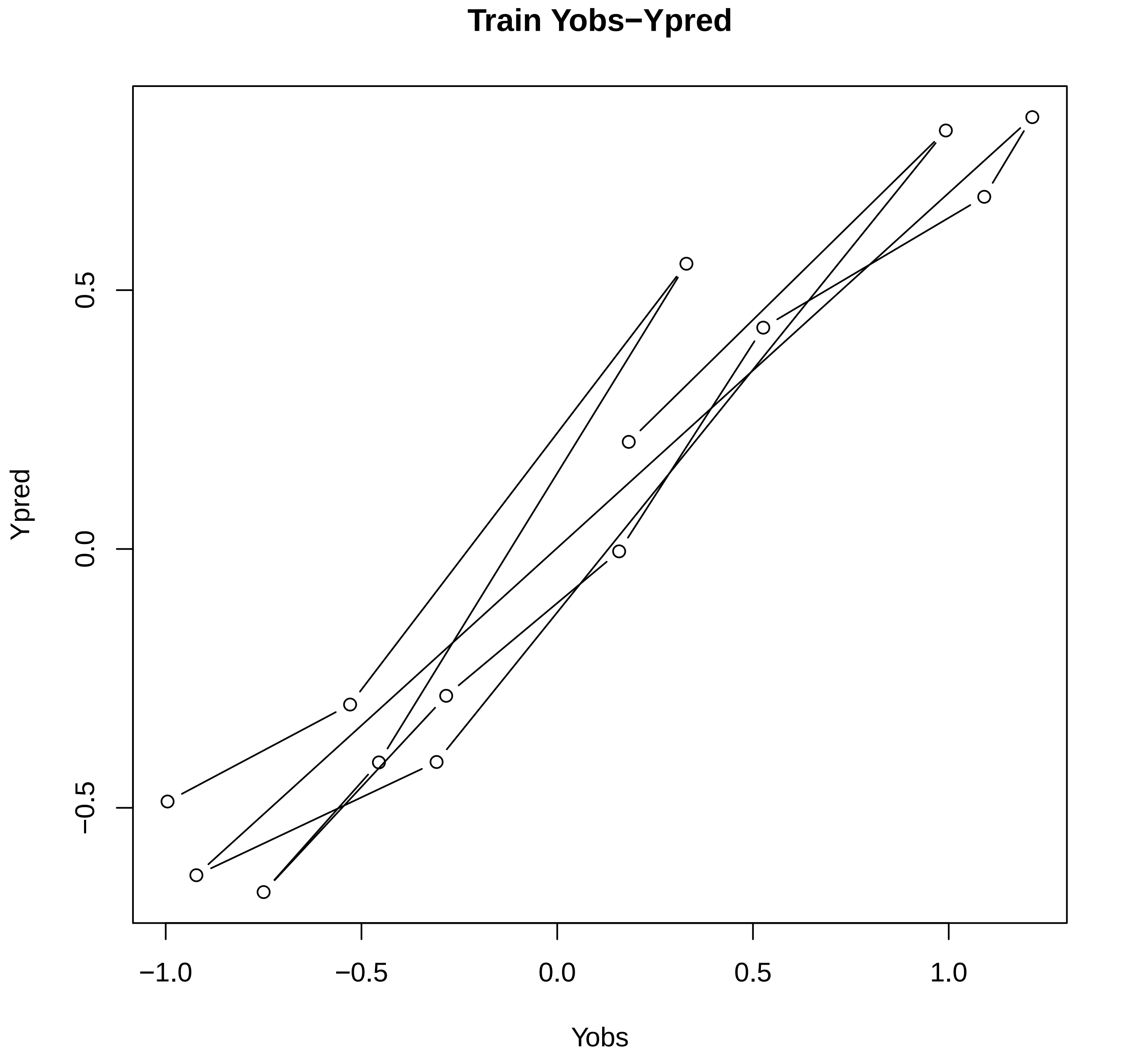
Train Yobs−Ypred
Ypred
Yobs
0.5
0.0
−0.5
−1.0
−0.5
0.0
0.5
1.0

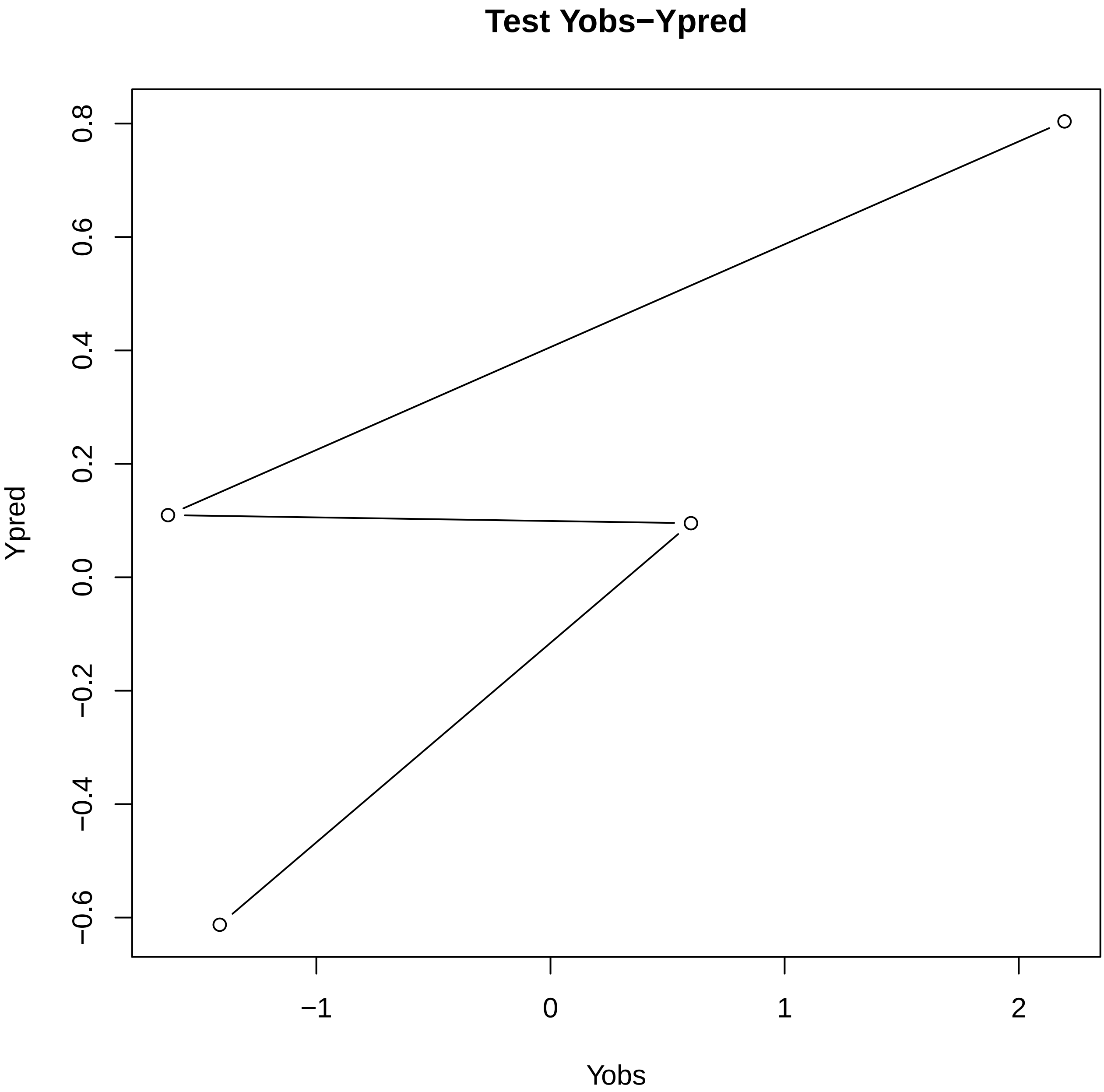

Test Yobs−Ypred
Ypred
0.8
0.6
0.4
0.2
0.0
−0.2
−0.4
−0.6
−1
0
1
2
Yobs

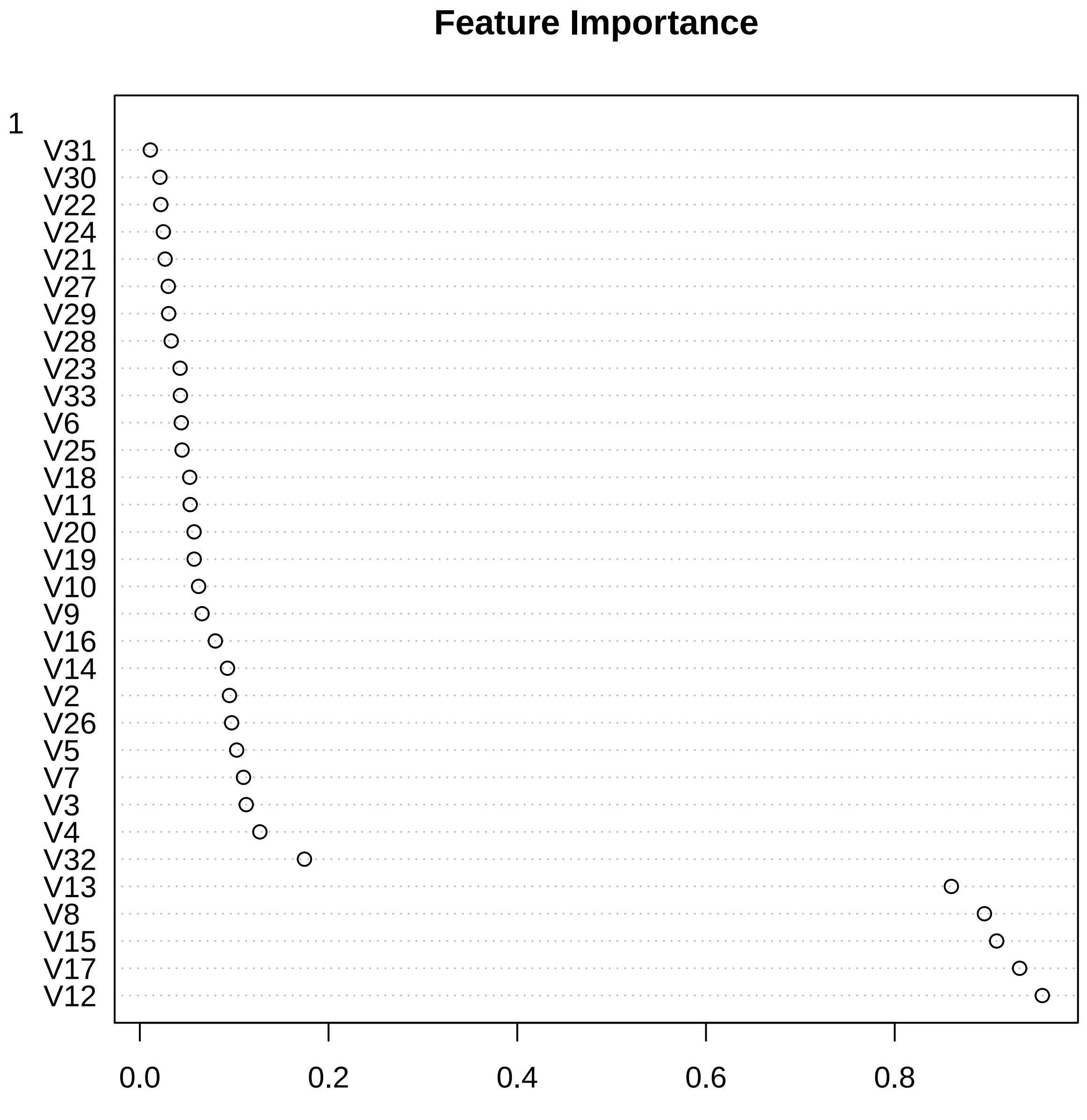

Feature Importance
1
V31
V30
V22
V24
V21
V27
V29
V28
V23
V33
V6
V25
V18
V11
V20
V19
V10
V9
V16
V14
V2
V26
V5
V7
V3
V4
V32
V13
V8
V15
V17
V12
0.0
0.2
0.4
0.6
0.8

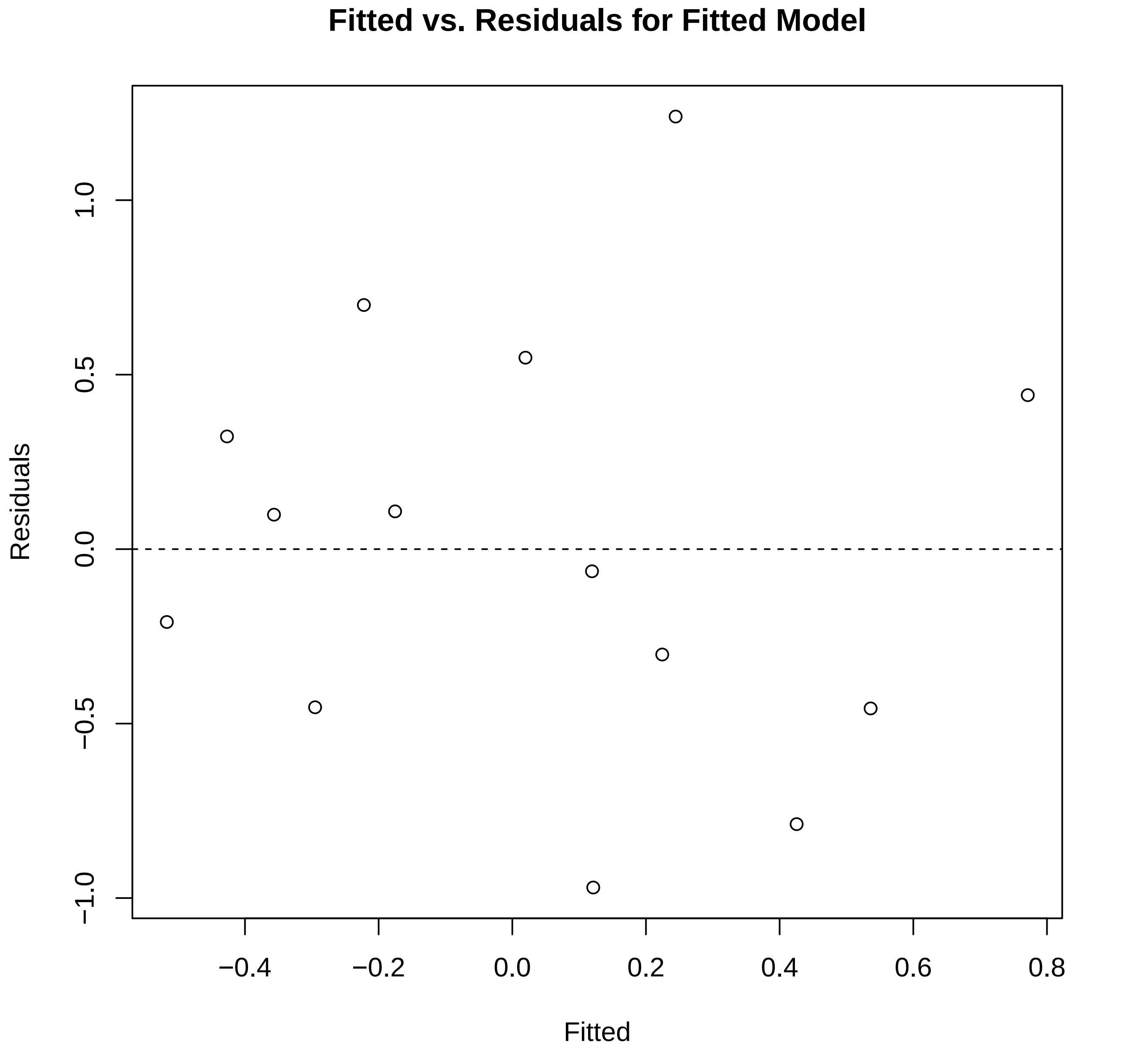

Fitted vs. Residuals for Fitted Model
Residuals
1.0
0.5
0.0
−0.5
−1.0
−0.4
−0.2
0.0
0.2
0.4
0.6
0.8
Fitted
